# Racial, Ethnic, and Gender Disparities in Awareness of Preexposure Prophylaxis Among HIV-Negative Heterosexually Active Adults at Increased Risk for HIV Infection — 23 Urban Areas, United States, 2019

**DOI:** 10.15585/mmwr.mm7047a3

**Published:** 2021-11-26

**Authors:** Amy R. Baugher, Lindsay Trujillo, Dafna Kanny, Jincong Q. Freeman, Terence Hickey, Catlainn Sionean, Ebony Respress, Johanna Chapin Bardales, Ruthanne Marcus, Teresa Finlayson, Cyprian Wejnert, National HIV Behavioral Surveillance Study Group, National HIV Behavioral Surveillance Study Group, Yingbo Ma, Hugo Santacruz, Ekow Kwa Sey, Adam Bente, Anna Flynn, Sheryl Williams, Willi McFarland, Desmond Miller, Danielle Veloso, Alia Al-Tayyib, Daniel Shodell, Irene Kuo, Jenevieve Opoku, Monica Faraldo, David Forrest, Emma Spencer, David Melton, Jeff Todd, Pascale Wortley, Antonio D. Jimenez, David Kern, Irina Tabidze, Narquis Barak, Jacob Chavez, William T. Robinson, Colin Flynn, Danielle German, Monina Klevens, Conall O’Cleirigh, Shauna Onofrey, Vivian Griffin, Emily Higgins, Corrine Sanger, Abdel R. Ibrahim, Corey Rosmarin-DeStafano, Afework Wogayehu, Meaghan Abrego, Bridget J. Anderson, Ashley Tate, Sarah Braunstein, Sidney Carrillo, Alexis Rivera, Lauren Lipira, Timothy W. Menza, E. Roberto Orellana, Tanner Nassau, Jennifer Shinefeld, Kathleen A. Brady, Sandra Miranda De León, María Pabón Martínez, Yadira Rolón-Colón, Meredith Brantley, Monica Kent, Jack Marr, Jie Deng, Margaret Vaaler, Salma Khuwaja, Zaida Lopez, Paige Padgett, Jennifer Kienzle, Toyah Reid, Brandie Smith, Sara Glick, Tom Jaenicke, Jennifer Reuer

**Affiliations:** ^1^Division of HIV Prevention, National Center for HIV, Viral Hepatitis, STD, and TB Prevention, CDC; ^2^ICF International, Fairfax, Virginia; ^3^Oak Ridge Institute for Science and Education, Oak Ridge, Tennessee.; Los Angeles, California; Los Angeles, California; Los Angeles, California; San Diego, California; San Diego, California; San Diego, California; San Francisco, California; San Francisco, California; San Francisco, California; Denver, Colorado; Denver, Colorado; Washington, D.C.; Washington, D.C.; Miami, Florida; Miami, Florida; Miami, Florida; Atlanta, Georgia; Atlanta, Georgia; Atlanta, Georgia; Chicago, Illinois; Chicago, Illinois; Chicago, Illinois; New Orleans, Louisiana; New Orleans, Louisiana; New Orleans, Louisiana; Baltimore, Maryland; Baltimore, Maryland; Boston, Massachusetts; Boston, Massachusetts; Boston, Massachusetts; Detroit, Michigan; Detroit, Michigan; Detroit, Michigan; Newark, New Jersey; Newark, New Jersey; Newark, New Jersey; Nassau and Suffolk counties, New York; Nassau and Suffolk counties, New York; Nassau and Suffolk counties, New York; New York, New York; New York, New York; New York, New York; Portland, Oregon; Portland, Oregon; Portland, Oregon; Philadelphia, Pennsylvania; Philadelphia, Pennsylvania; Philadelphia, Pennsylvania; San Juan, Puerto Rico; San Juan, Puerto Rico; San Juan, Puerto Rico; Memphis, Tennessee; Memphis, Tennessee; Memphis, Tennessee; Dallas, Texas; Dallas, Texas; Houston, Texas; Houston, Texas; Houston, Texas; Virginia Beach, Virginia; Virginia Beach, Virginia; Virginia Beach, Virginia; Seattle, Washington; Seattle, Washington; Seattle, Washington

In 2019, heterosexual sex accounted for 23% of new HIV diagnoses in the United States and six dependent areas (*1*). Although preexposure prophylaxis (PrEP) can safely reduce the risk for HIV infection among heterosexual persons, this group is underrepresented in PrEP research (*2*). CDC analyzed National HIV Behavioral Surveillance (NHBS) data to describe PrEP awareness among heterosexually active adults in cities with high HIV prevalence. Overall, although 32.3% of heterosexually active adults who were eligible were aware of PrEP, <1% used PrEP. Racial, ethnic, and gender disparities were identified, with the lowest awareness of PrEP among residents of Puerto Rico (5.8%) and Hispanic or Latino (Hispanic) men (19.5%) and women (17.6%). Previous studies have found that heterosexual adults are interested in taking PrEP when they are aware of it (*3*); tailoring PrEP messaging, including Spanish-language messaging, to heterosexual adults, might increase PrEP awareness and mitigate disparities in use.

The 2019 NHBS cycle included face-to-face interviews and HIV testing among eligible* heterosexually active adults in 23 urban areas with high HIV prevalence. Detailed information about the 2019 NHBS cycle, including sampling methods, have been described in the CDC’s HIV Surveillance Special Report 26 (*4*). This analysis was limited to participants who received a negative HIV test result and reported low income^†^; NHBS uses low income as a proxy for increased risk for acquiring HIV through heterosexual sex (*4*). PrEP awareness was defined as having ever heard of PrEP. Not all participants might be candidates for PrEP use; however, PrEP awareness might be beneficial to persons regardless of their own PrEP eligibility.^§^ Demographic and social determinants of health^¶^ differences in PrEP awareness were assessed using log-linked Poisson regression models** with generalized estimating equations to calculate adjusted prevalence ratios (aPRs) and 95% CIs. PrEP use could not be analyzed or stratified because use prevalence was <1%. Analyses were conducted using SAS (version 9.4; SAS Institute). This activity was reviewed by CDC and was conducted consistent with applicable federal law and CDC policy.^††^

Among 9,359 total participants, 3,026 (32.3%) were aware of PrEP, including 1,221 (29.2%) men and 1,805 (34.8%) women (Table). Overall, 19.5% of Hispanic, 24.2% of White, and 31.9% of Black heterosexually active men were aware of PrEP (Figure). Overall, 17.6% of Hispanic, 32.7% of White, and 40.3% of Black heterosexually active women were aware of PrEP. Awareness of PrEP was lower among Hispanic women than among both Hispanic men and other racial/ethnic groups of women. Lower PrEP awareness was found among uninsured participants (26.4%) than among insured participants (34.2%) (aPR = 0.76) and among participants without a usual source of care (29.1%) than among those with a usual source of care (34.3%) (aPR = 0.82) (Table). PrEP awareness was lower among participants born in Puerto Rico (8.2%; aPR = 0.57) or Mexico (12.6%; aPR = 0.57) than among participants born in the 50 United States and the District of Columbia (35.1%). Non–U.S.-born participants who did not speak English well reported lower PrEP awareness than did U.S.-born participants (6.5% versus 35.2%; aPR = 0.26); higher PrEP awareness was reported with increasing English proficiency. Participants residing in Puerto Rico reported lower PrEP awareness than did participants residing in the South U.S. Census Region (5.8% versus 36.0%; aPR = 0.14).

**TABLE:** Preexposure prophylaxis awareness among HIV-negative* heterosexually active men and women who are at increased risk for HIV infection (N = 9,359) — National HIV Behavioral Surveillance, 23 urban areas, United States, 2019

Characteristic	Awareness of PrEP no. (%)^†^	Adjusted prevalence ratio (95% CI)^§^
**Overall**	**3,026 (32.3)**	**NA**
**Sex**		
Men	1,221 (29.2)	0.79 (0.74–0.85)
Women	1,805 (34.8)	Ref
**Race/Ethnicity** ^¶^		
AI/AN	23 (39.7)	1.10 (0.81–1.48)
Asian	8 (47.1)	1.39 (0.94–2.05)
Black	2,322 (36.4)	Ref
Hispanic	382 (18.4)	0.69 (0.60–0.79)
NH/OPI	11 (33.3)	1.02 (0.65–1.61)
White	122 (29.5)	0.87 (0.73–1.03)
Multiple races	147 (40.6)	1.14 (1.02–1.28)
**Age group, yrs**		
18–29	968 (30.3)	1.10 (1.02–1.19)
30–39	826 (36.4)	1.31 (1.21–1.42)
40–49	600 (34.2)	1.19 (1.08–1.31)
50–60	632 (29.5)	Ref
Federal poverty level**		
At or below federal poverty level	2,391 (31.4)	0.86 (0.80–0.92)
Above federal poverty level	635 (36.4)	Ref
**Education**		
Less than high school	707 (29.3)	0.66 (0.56–0.76)
High school diploma or equivalent	1,414 (30.2)	0.68 (0.59–0.79)
Some college or technical degree	803 (39.6)	0.92 (0.79–1.06)
College degree or more	101 (42.6)	Ref
**Currently have health insurance**		
Yes	2,427 (34.2)	Ref
No	586 (26.4)	0.76 (0.70–0.83)
**Have a usual source of health care**		
Yes	2,002 (34.3)	Ref
No	1,001 (29.1)	0.82 (0.78–0.87)
**Place of birth** ^††^		
50 U.S. states or District of Columbia	2,896 (35.1)	Ref
Puerto Rico	42 (8.2)	0.57 (0.47–0.69)
Mexico	22 (12.6)	0.57 (0.42–0.77)
Central America (other)	8 (5.8)	0.21 (0.10–0.45)
Cuba	9 (15.5)	0.39 (0.18–0.84)
Caribbean (other)	20 (23.0)	0.67 (0.43–1.06)
South America	3 (6.8)	0.26 (0.12–0.56)
Europe	5 (20.0)	0.67 (0.27–1.64)
Asia	10 (45.5)	1.30 (0.78–2.15)
Africa	8 (26.7)	0.83 (0.45–1.51)
**Years lived in United States^§§^**		
U.S.-born	2,895 (35.2)	Ref
Non–U.S.-born, >5 yrs	94 (17.4)	0.58 (0.48–0.71)
Non–U.S.-born, ≤5 yrs	9 (8.4)	0.33 (0.22–0.50)
**Proficiency in English** ^§§^		
U.S.-born	2,895 (35.2)	Ref
Non–U.S.-born, speaks English well	86 (22.3)	0.69 (0.57–0.84)
Non–U.S.-born, does not speak English well	17 (6.5)	0.26 (0.19–0.37)
**U.S. Census region of current residence** ^¶¶^		
Northeast	717 (33.2)	0.97 (0.82–1.15)
Midwest	310 (37.6)	1.21 (1.02–1.43)
South	1,364 (36.0)	Ref
West	607 (28.8)	0.73 (0.62–0.88)
Puerto Rico	28 (5.8)	0.14 (0.11–0.17)

**Abbreviations:** AI/AN = American Indian/Alaska Native; HHS = U.S. Department of Health and Human Services; NA = not applicable; NHBS = National HIV Behavioral Surveillance; NH/OPI = Native Hawaiian/Other Pacific Islander; Ref = referent group.

* Participants with a valid negative NHBS HIV test result.

^†^ Row percentages of persons who had ever heard of PrEP: “Preexposure prophylaxis, or PrEP, is an antiretroviral medicine, such as Truvada, taken for months or years by a person who is HIV-negative to reduce the risk for acquiring HIV. Before today, have you ever heard of PrEP?”

^§^ Log-linked Poisson regression models were adjusted for urban area and network size and clustered on recruitment chain.

^¶^ Hispanic persons could be of any race; all racial and ethnic groups are mutually exclusive.

** Poverty level was defined by the 2018 HHS Poverty Guidelines. https://aspe.hhs.gov/2018-poverty-guidelines

^††^ Frequently reported countries or territories were reported separately; other countries were grouped by geographic region.

^§§^ Participants residing in San Juan, Puerto Rico were excluded. English language proficiency was measured using HHS data collection standards. https://aspe.hhs.gov/reports/hhs-implementation-guidance-data-collection-standards-race-ethnicity-sex-primary-language-disability-0

^¶¶^ *Northeast:* Boston, Massachusetts; Nassau and Suffolk counties, New York; New York City, New York; Newark, New Jersey; and Philadelphia, Pennsylvania. *Midwest:* Chicago, Illinois and Detroit, Michigan. *South*: Atlanta, Georgia; Baltimore, Maryland; Dallas, Texas; Houston, Texas; Miami, Florida; New Orleans, Louisiana; and Washington, DC. *West:* Denver, Colorado; Los Angeles, California; San Diego, California; San Francisco, California; and Seattle, Washington. *Puerto Rico:* San Juan, Puerto Rico.

**FIGURE:**
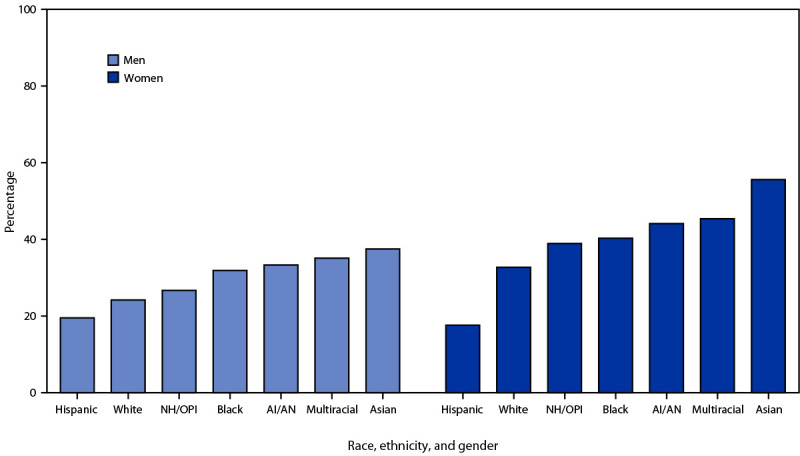
Percentage of HIV-negative heterosexually active men and women who had heard of preexposure prophylaxis (N = 9,359), by race, ethnicity,* and gender — National HIV Behavioral Surveillance, 23 urban areas, United States, 2019 **Abbreviations**: AI/AN = American Indian/Alaska Native; NH/OPI = Native Hawaiian/Other Pacific Islander. * Hispanic persons could be of any race; other race groups were non-Hispanic. NH/OPI, AI/AN, and Asian men and women included ≤15 persons per group.

## Discussion

In 2019, PrEP use among eligible heterosexually active adults was negligible (<1%), and approximately one in three heterosexually active adults was aware of PrEP. Overall, men reported lower PrEP awareness than did women. Awareness of PrEP was particularly low among Hispanic persons, with approximately one in six Hispanic women and approximately one in five Hispanic men having heard of PrEP. Awareness of PrEP was also low among persons who were not born in the United States, did not speak English well, or who resided in Puerto Rico. Given the high prevalence of HIV infection among Black persons, it is notable that their PrEP awareness was relatively higher than that among White or Hispanic persons. This might be attributable to HIV prevention campaigns tailored toward Black persons.

Awareness of PrEP and its potential to prevent sexually transmitted HIV infection is needed to end the HIV epidemic in the United States. PrEP use has the potential to reduce persistent racial, ethnic, and gender disparities in HIV infection observed among heterosexually active adults. In 2019, Black and Hispanic women accounted for 60.0% and 18.6% of new HIV diagnoses among women, respectively. Black and Hispanic men accounted for 61.2% and 20.3% of new HIV diagnoses attributed to heterosexual sex among men, respectively (*1*).

PrEP awareness might be low for multiple reasons, including limited tailored communications and infrequent patient-provider discussions about PrEP. In addition, few PrEP campaigns focus on heterosexual adults, particularly Hispanic persons. Although some prevention resources for heterosexual adults are inclusive of PrEP,^§§^ most PrEP campaigns focus on men who have sex with men (MSM), which can reinforce stereotypes that PrEP is only intended for MSM (*5*). Because of stigma and gender norms, these stereotypes might interfere with marketing HIV prevention to some heterosexual Hispanic adults (*6*). Previous studies have indicated that heterosexual adults might not perceive themselves as being at risk for HIV infection or as candidates for PrEP (*7*).

Campaigns and interventions providing PrEP information and resources designed for heterosexual adults are limited. In 2021, CDC launched #ShesWell,^¶¶^ which promotes PrEP among women; however, there are currently no national PrEP campaigns focused on all heterosexual adults at increased risk for HIV acquisition or heterosexual Hispanic men or women. Existing HIV interventions, such as Sister to Sister,*** which is geared toward Black women aged 18–45 years and implemented in primary care provider settings, could be expanded and tailored to other groups. 

Although some PrEP resources are available in Spanish,^†††^ few PrEP materials are designed for specific groups of heterosexually active adults, including Hispanic persons, women, persons born outside the United States, and persons residing in Puerto Rico. Studies have also found that persons at high risk for HIV infection need messaging specifically customized for their population (*8*). Culturally competent PrEP materials and media campaigns geared toward heterosexual adults, including products tailored for heterosexual Hispanic persons, communicated through channels that might better reach Hispanic audiences, and which are available in Spanish, could increase PrEP awareness and use in this group (*6*). In addition, personalized PrEP campaign messaging might help heterosexual adults envision how PrEP could benefit them (*9*).

PrEP awareness might also be low because, despite CDC guidance, health care providers often do not discuss PrEP with heterosexual patients at increased risk for acquiring HIV (*10*). Primary care physicians and obstetricians and gynecologists can use routine visits and HIV and sexually transmitted infection testing encounters to educate their heterosexual patients about PrEP and screen for PrEP eligibility (*5*). Providers can assess barriers to PrEP use at multiple levels, including individual (e.g., side effects), interpersonal (e.g., judgment from others), community (e.g., caregiving duties), and structural (e.g., insurance and unstable housing) (*10*). Alternative PrEP options are emerging that allow ease of use, convenience, and confidentiality (e.g., vaginal rings and long-acting injectables) (*10*). Alternative modalities might broaden the appeal of PrEP among women (*2*) and encourage patient-provider discussions as part of sexual health assessments.

The findings in this report are subject to at least five limitations. First, data are not representative of all heterosexual men and women because the sample consists of low-income persons residing in 23 urban areas. Second, self-reported data are subject to recall and social desirability biases. Third, although awareness of PrEP is low, it is unknown whether participants had been exposed to existing PrEP campaigns. Fourth, PrEP awareness does not necessarily imply accurate knowledge or positive attitudes about PrEP. Persons might have heard of PrEP but might not be aware of their own eligibility. Finally, NHBS data are cross-sectional and do not support causal inference.

PrEP awareness among heterosexually active adults in the United States is low, especially among Hispanic men and women and persons residing in Puerto Rico. Although PrEP is not recommended for everyone, increasing awareness of PrEP in the general population could shape public attitudes and reduce stigma associated with PrEP and HIV (*8*,*9*). In addition to tailored, culturally appropriate campaigns for heterosexually active adults at risk for HIV infection, there are opportunities to increase awareness and use of PrEP through increased screening and patient-provider communication. Along with other preventive measures, increasing PrEP use among heterosexual persons is needed to end the HIV epidemic in the United States.

**Summary**

**What is already known about this topic?**

Heterosexual sex accounts for 23% of new HIV diagnoses annually. Heterosexual adults are underrepresented in preexposure prophylaxis (PrEP) research and campaigns. Increasing PrEP awareness and use in this population is needed to prevent HIV transmission and end the HIV epidemic in the United States.

**What is added by this report?**

PrEP awareness (32.3%) and use (<1%) among heterosexually active adults in high-prevalence cities is low, especially among Hispanic or Latino men and women (19.5% and 17.6%, respectively) and persons residing in Puerto Rico (5.8%).

**What are the implications for public health practice?**

Tailored PrEP campaigns and routine screening can increase PrEP awareness and use among heterosexual adults, particularly among Hispanic persons.

## Footnotes

* Eligibility to participate in the study included never having had male-to-male sexual contact, aged 18–60 years, no previous participation in NHBS during 2019, residence in a participating urban area, ability to complete the survey in English or Spanish, report of vaginal or anal sex with an opposite-sex partner in the past 12 months, and never having injected drugs.

† Low income is defined as income at or below 150% of the federal poverty level, adjusted for geographic cost of living differences.

§
https://www.cdc.gov/hiv/pdf/risk/prep/cdc-hiv-prep-guidelines-2017.pdf

¶ Race and Hispanic ethnicity were presented separately, consistent with U.S. Department of Health and Human Services (HHS) and Office of Management and Budget standards for race/ethnicity categorization. All racial groups are mutually exclusive (https://aspe.hhs.gov/reports/hhs-implementation-guidance-data-collection-standards-race-ethnicity-sex-primary-language-disability-0). Poverty was defined as income at or below the 2018 HHS poverty guidelines (https://aspe.hhs.gov/topics/poverty-economic-mobility/poverty-guidelines/prior-hhs-poverty-guidelines-federal-register-references/2018-poverty-guidelines). Place of birth was defined as country or territory of birth. Participants born in Puerto Rico were presented separately from participants born in the 50 U.S. states and the District of Columbia. Frequently reported countries or territories were reported individually; other countries were grouped by global region. Years of U.S. residence was defined as the number of years the participant had resided in the United States. Participants residing in Puerto Rico were excluded from analysis of years of U.S. residency because of the design of the survey instrument. The South U.S. Census Region was selected as the referent Census region because CDC’s Division of HIV Prevention’s Strategic Plan prioritized the South (https://www.cdc.gov/hiv/pdf/dhap/cdc-hiv-dhap-external-strategic-plan.pdf). Participants were asked to self-rate their English proficiency based on the HHS data standard for primary language (https://aspe.hhs.gov/reports/hhs-implementation-guidance-data-collection-standards-race-ethnicity-sex-primary-language-disability-0). All U.S.-born participants were proficient in English; non–U.S.-born participants were analyzed by English proficiency level. Participants residing in Puerto Rico were excluded from analysis of English proficiency because Spanish is the predominant language in Puerto Rico. Usual source of care was defined as having a usual source for health care other than a hospital emergency department.

** Models were adjusted for urban area and network size and clustered on recruitment chain.

†† 45 C.F.R. part 46.102(l)(2), 21 C.F.R. part 56; 42 U.S.C. Sect. 241(d); 5 U.S.C. Sect. 552a; 44 U.S.C. Sect. 3501 et seq.

§§
https://www.hiv.gov/federal-response/ending-the-hiv-epidemic/prep-program

¶¶
https://www.cdc.gov/stophivtogether/sheiswell/index.html

***
https://www.cdc.gov/hiv/effective-interventions/prevent/sister-to-sister

†††
https://www.cdc.gov/stophivtogether/spanish/index.html
